# Identifying luminal and basal mammary cell specific genes and their expression patterns during pregnancy

**DOI:** 10.1371/journal.pone.0267211

**Published:** 2022-04-29

**Authors:** Zhan Dong Li, Xiangtian Yu, Zi Mei, Tao Zeng, Lei Chen, Xian Ling Xu, Hao Li, Tao Huang, Yu-Dong Cai

**Affiliations:** 1 College of Biological and Food Engineering, Jilin Engineering Normal University, Changchun, China; 2 Shanghai Jiao Tong University Affiliated Sixth People’s Hospital, Shanghai, China; 3 Shanghai Institute of Nutrition and Health, Chinese Academy of Sciences, Shanghai, China; 4 Bio-Med Big Data Center, CAS Key Laboratory of Computational Biology, Shanghai Institute of Nutrition and Health, University of Chinese Academy of Sciences, Chinese Academy of Sciences, Shanghai, China; 5 College of Information Engineering, Shanghai Maritime University, Shanghai, China; 6 Guangdong AIB Polytechnic College, Guangzhou, China; 7 CAS Key Laboratory of Tissue Microenvironment and Tumor, Shanghai Institute of Nutrition and Health, University of Chinese Academy of Sciences, Chinese Academy of Sciences, Shanghai, China; 8 School of Life Sciences, Shanghai University, Shanghai, China; University of Salerno, ITALY

## Abstract

Mammary gland is present in all mammals and usually functions in producing milk to feed the young offspring. Mammogenesis refers to the growth and development of mammary gland, which begins at puberty and ends after lactation. Pregnancy is regulated by various cytokines, which further contributes to mammary gland development. Epithelial cells, including basal and luminal cells, are one of the major components of mammary gland cells. The development of basal and luminal cells has been observed to significantly differ at different stages. However, the underlying mechanisms for differences between basal and luminal cells have not been fully studied. To explore the mechanisms underlying the differentiation of mammary progenitors or their offspring into luminal and myoepithelial cells, the single-cell sequencing data on mammary epithelia cells of virgin and pregnant mouse was deeply investigated in this work. We evaluated features by using Monte Carlo feature selection and plotted the incremental feature selection curve with support vector machine or RIPPER to find the optimal gene features and rules that can divide epithelial cells into four clusters with different cell subtypes like basal and luminal cells and different phases like pregnancy and virginity. As representations, the feature genes *Cldn7*, *Gjb6*, *Sparc*, *Cldn3*, *Cited1*, *Krt17*, *Spp1*, *Cldn4*, *Gjb2* and *Cldn19* might play an important role in classifying the epithelial mammary cells. Notably, seven most important rules based on the combination of cell-specific and tissue-specific expressions of feature genes effectively classify the epithelial mammary cells in a quantitative and interpretable manner.

## Introduction

The mammary gland is an exocrine gland in mammals. It distinguishes mammals from all other animals and functions to produce milk to feed young offspring. The milk containing nutrients and antibodies is passed to the newborn through breastfeeding to ensure its development and growth. The mammary glands exist in both sexes, while male mammary glands are generally non-functional because of degeneration. Mammogenesis, which refers to the growth and development of the mammary gland, has different growth cycles, including puberty growth, pregnancy, lactation, and degeneration. Considering that the ectoderm forms a mammary gland, it resolves into a plate and the first one in the embryo occurs. The placodes descend into the underlying mesenchyme and produces the basic ductal structure of the glands present at birth, and the processes are regulated by interactions between epithelial and mesenchymal cells. Branch morphogenesis occurs at puberty, and this process requires growth hormone, estrogen, and IGF1 to produce a dust tree filled with a fat pad. During pregnancy, the combined action of progesterone and prolactin produces alveoli, which secretes milk during lactation. The lack of milk demand during weaning can lead to a degenerative process that restores the gland to its pre-pregnancy state [1]. During menopause, mammogenesis stops, and the breasts undergo atrophy.

Mammogenesis mainly occurs during gestation, and certain cytokines that maintain pregnancy play a crucial role in regulating mammary gland development during pregnancy. Some pregnancy hormones, such as estrogen and progesterone, are essential for mammary growth and maturation [2]. During pregnancy, the breast experiences significant growth and maturation in preparation for breastfeeding. Estrogen and progesterone levels increase greatly in a short time, reaching hundreds of times higher than the usual menstrual cycle levels during late pregnancy [3]. Estrogen and progesterone cause the anterior pituitary gland to secrete high levels of prolactin, which can reach 20 times than the normal menstrual cycle level [4].

Generally, the mammary epithelium cells consist of two main cell types, namely, basal and luminal cells. The mechanisms underlying the differentiation of mammary progenitors or their offspring into luminal and myoepithelial cells require further study [5]. Luminal cells or luminal progenitors can introduce alveolar epithelial cells during pregnancy, and this differentiation is driven by several factors, including transcription factor Gata-3,β3-integrin/CD61 [6]. The luminal cell compartment of the mouse mammary gland can be resolved into non-clonogenic estrogen receptor-positive (ER+) luminal cells, ER+ luminal progenitors, and estrogen receptor-negative (ER-) liminal progenitors. All luminal progenitor cells in human and mouse have pluripotency that can produce offspring differentiating into all mammary cell types at low frequencies. The luminal cell compartment in the mammary epithelium becomes more heterogeneous during differentiation and proliferation, because varied levels of cellular plasticity can be identified in luminal progenitor cell [7]. Moreover, basal cells are important compartment of mammary epithelium; they are contractile and contact the basement membrane. Cells of the basal lineage may have a critical tumor suppressive role in preventing the progression of ductal carcinoma *in situ* to invasive ductal carcinoma [2]. However, some transcription factors are involved in the differentiation of a basal progenitor into a myoepithelial cell. Transcription factors such as Slug, Smad 3, and Notch signaling are related to the basal phenotype [8].

Actually, the development of epithelial mammary cells, which are consisted of basal and luminal, significantly differ at different stages. They develop differently in the phases of virginity and pregnancy, and research is lacking about the genes that can be used to classify epithelial mammary cells. In the present work, a deep investigation on the single-cell sequencing data on mammary epithelia cells of virgin and pregnant mouse was conducted. The feature genes were analyzed by Monte Carlo Feature Selection (MCFS) [9]. The result was fed into the incremental feature selection (IFS) [10], incorporating support vector machine (SVM) [11] or repeated incremental pruning to produce error reduction (RIPPER) [12] as classification algorithm to determine essential gene features and rules that are significant to divide the mammary cells into four types, including basal and luminal cell in the phases of pregnancy and virginity. According to these feature genes and rules, *Cldn7*, *Gjb6*, *Sparc*, *Cldn3*, *Cited1*, *Krt17*, *Spp1*, *Cldn4*, *Gjb2* and *Cldn19* might play an important role in classifying the epithelial mammary cells. In addition, we determined the seven most important rules for classifying the epithelial mammary cells in a quantitative manner by the combination of cell- and tissue-specific expressions of feature genes.

## Materials and methods

### Dataset

Single-cell sequencing data on mammary epithelia cells of virgin and pregnant mouse were downloaded from the Gene Expression Omnibus (GEO) database (https://www.ncbi.nlm.nih.gov/geo/query/acc.cgi?acc=GSE110371) [13]. A total of 102 PB (basal mammary cell, pregnant day 12), 49 PL (luminal mammary cell, pregnant day 12), 34 VB (basal mammary cell, virgin), and 54 VL (luminal mammary cell, virgin) cells were observed. The expression data of 18,850 genes were analyzed using the Illumina HiSeq 2500 platform. We aimed to explore the mammary epithelia changes after pregnancy.

### Monte Carlo feature selection

MCFS [9] is a widely used and stable feature selection method in many biological studies, and this method is based on random sampling approach. Briefly, in an MCFS analysis, many decision trees are built on one bootstrap sample-set with a randomly selected feature subset (e.g., feature subset with *m* features are selected from the raw *M* features, and *m* is far smaller than *M*). For each feature subset, *p* decision trees can be obtained through training and evaluation on bootstrapping sets consisting of data with this feature subset. When this procedure is iterated for *T* times, *p* × *T* decision trees can be produced, and relative importance (RI) can be calculated based on the contribution of features in each decision tree classifier constructed by the above steps. In detail, the formula for calculating the RI score of a feature *g* is as follows:

$$RI_{g}=\sum_{\tau =1}^{p\times T}{\left(wAcc\right)}^{u}IG(n_{g}(\tau )){\left(\frac{no.in\;n_{g}(\tau )}{no.in\;\tau }\right)}^{v},$$ (1)

where wAcc is the weighted accuracy generated by the average sensitivity of all decision classes, *n*_*g*_(*τ*) is the node that participate in the decision tree *τ* using feature *g*, *IG*(*n*_*g*_(*τ*)) is the information gain of *n*_*g*_(*τ*), and *no*.*in τ* and *no*.*in n*_*g*_(*τ*) denote the size of sample data in the decision tree *τ* and the number of training sample data in node *n*_*g*_(*τ*), respectively. Besides, *u* and *v* are the corresponding weighting factors for adjusting the contributions of different optimal targets. Clearly, features with high RI scores are more important than those with low RI scores. Accordingly, a feature list can be built by the decreasing order of features’ RI scores. In this study, this list was denoted by *F*.

To execute the MCFS, we downloaded its program at http://www.ipipan.eu/staff/m.draminski/mcfs.html. For convenience, it was performed with its default parameters.

### Incremental feature selection

After generating a ranked feature list by MCFS, IFS can be used to further determine a series of key features to precisely distinguish different sample groups (e.g., cell types) [10]. In detail, a series of feature subsets with an interval of 10 was generated from the ranked feature list *F* by MCFS. We generated *m* feature subsets $F_{1}^{1},F_{2}^{1},\ldots ,F_{m}^{1}$, where the *i-*th feature subset contains the top-ranked 10×*i* features $F_{i}^{1}=[f_{1},f_{2},\ldots ,f_{i\times 10}]$. Then, on each feature subset, a classifier was built with a given classification algorithm and samples represented by features in this subset. The classifier was further tested by 10-fold cross-validation [14]. After all classifiers have been tested, the classifier providing the best performance can be identified. This classifier was called the optimum classifier and features used in this classifier were termed as optimum features.

### Classification algorithm

To execute IFS method, a classification algorithm was necessary. Here, we employed two classic algorithms: SVM [11] and RIPPER [12].

#### Support vector machine

SVM is a popular and useful machine learning algorithm for supervised learning [11, 15–21]. It adopts kernel techniques (e.g., Gaussian kernels) based on statistical learning theory, and it maps raw data from low-dimensional nonlinear feature space to high-dimensional linear feature space, so that, a hyperplane (e.g. a linear function) in the high-dimensional space with the largest margin can be used to separate the samples in the training data set. In the present work, the tool “SMO” implemented in Weka [22] software was employed for the building of SVM with the default parameter setting. Although SVM is quite powerful, its classification principles are very hard for human to understand. Thus, it is proper to set up efficient tools for classifying mammary epithelia cells rather than uncover the mammary epithelia changes after pregnancy. In view of this, another rule learning algorithm, RIPPER, was also employed.

#### Rule learning

In addition to the above “black-box” algorithm, SVM, we also used RIPPER to learn decision classification rules for model interpretation. In RIPPER, each rule is represented as an IF-ELSE statement. For instance, gene1 >2.5 and gene2 <10 indicate a basal cell. These learned rules can be used to make human-readable predictions for new samples. In the present work, the tool “JRip” implemented in Weka [22] was used for extracting RIPPER rules.

### Performance measurement

To access the performance of particular classification models, we adopted Matthew’s correlation coefficient (MCC) [23, 24] as an evaluation metric. MCC was first designed for binary classification and is widely used in the field of bioinformatics [15, 25–29]. In the present work, considering the multi-classification model learned, the multi-class version of MCC was applied [24], and this parameter is calculated using the following formula:

$$MCC=\frac{\text{cov}(X,Y)}{\sqrt{\text{cov}(X,X)\text{cov}(Y,Y)}},$$ (2)

where *X* is a 0–1 matrix representing the predicted category of each sample, *Y* is also a 0–1 matrix representing the true classes of all samples, and cov(·,·) is the covariance of the two matrices. The multi-class version of MCC has been broadly employed [30–32] so that it is still referred to as MCC in the following analysis for convenience.

In addition, we also calculated other measurements to fully evaluate the performance of classifiers, including overall accuracy and individual accuracy on each class. The overall accuracy is defined as the proportion of correctly predicted samples among all samples, whereas the individual accuracy on one class is the proportion of correctly predicted samples in this class among all samples in such class.

## Results and discussion

**Fig 1:**
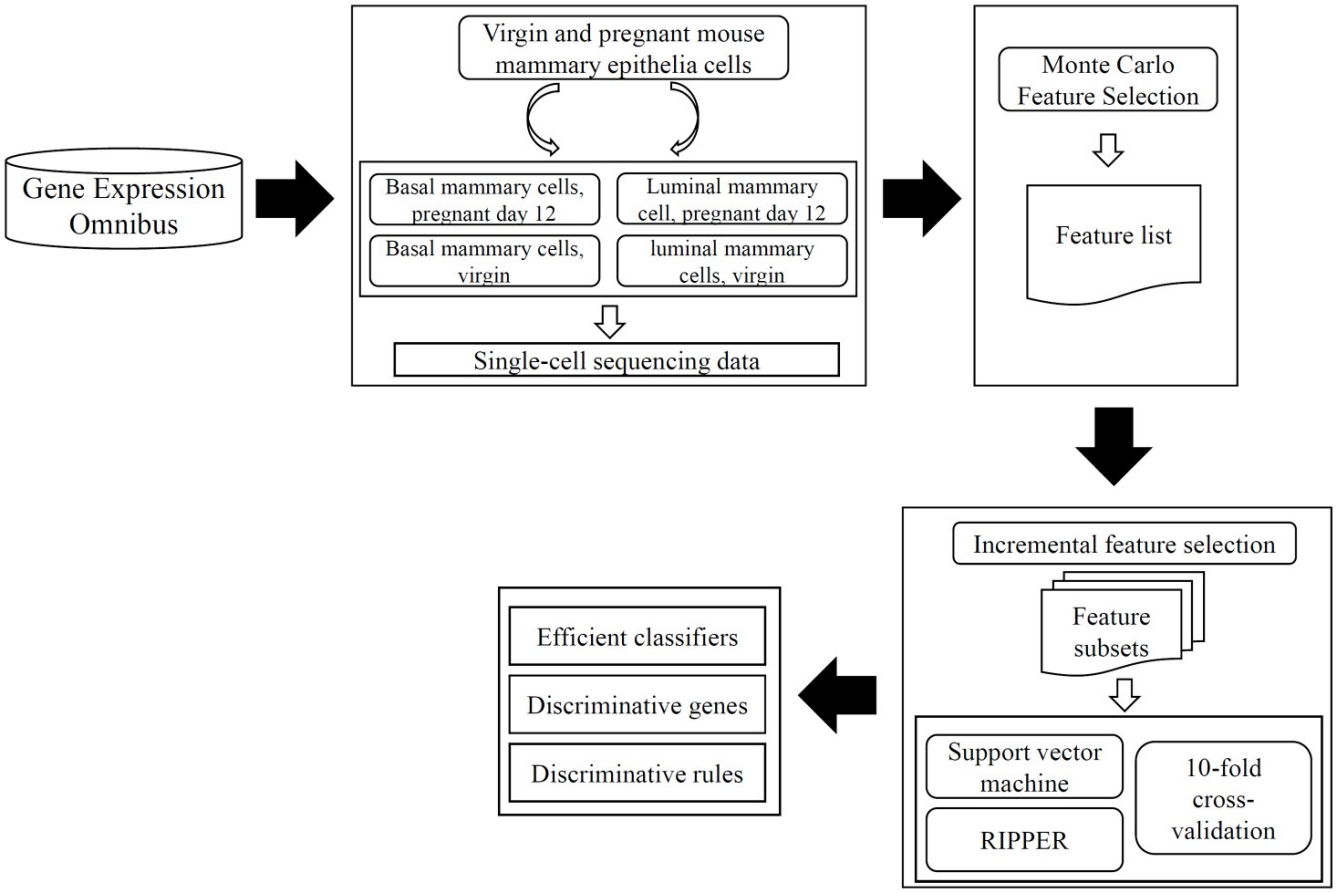
Entire procedures to analyze the single-cell sequencing data for virgin and pregnant mouse mammary epithelia cells. The data is retrieved from Gene Expression Omnibus. Powerful Monte Carlo Feature Selection is applied on such data, resulting in a feature list. This list is fed into incremental feature selection, incorporating two classification algorithms, to build efficient classifiers and extract discriminative genes and rules.

In this study, a deep computational analysis was conducted on the single-cell sequencing data for virgin and pregnant mouse mammary epithelia cells. The entire procedures are shown in Fig 1. This section gave the detailed analysis results and further discussions.

### Results of MCFS method

According to Fig 1, the MCFS method was first applied to the single-cell sequencing data. It analyzed the importance of each feature by assigning it a RI score, which is provided in S1 Table. After that, all features were sorted by the decreasing order of their RI scores in a feature list, which is also provided in S1 Table.

### Results of IFS with SVM

**Fig 2:**
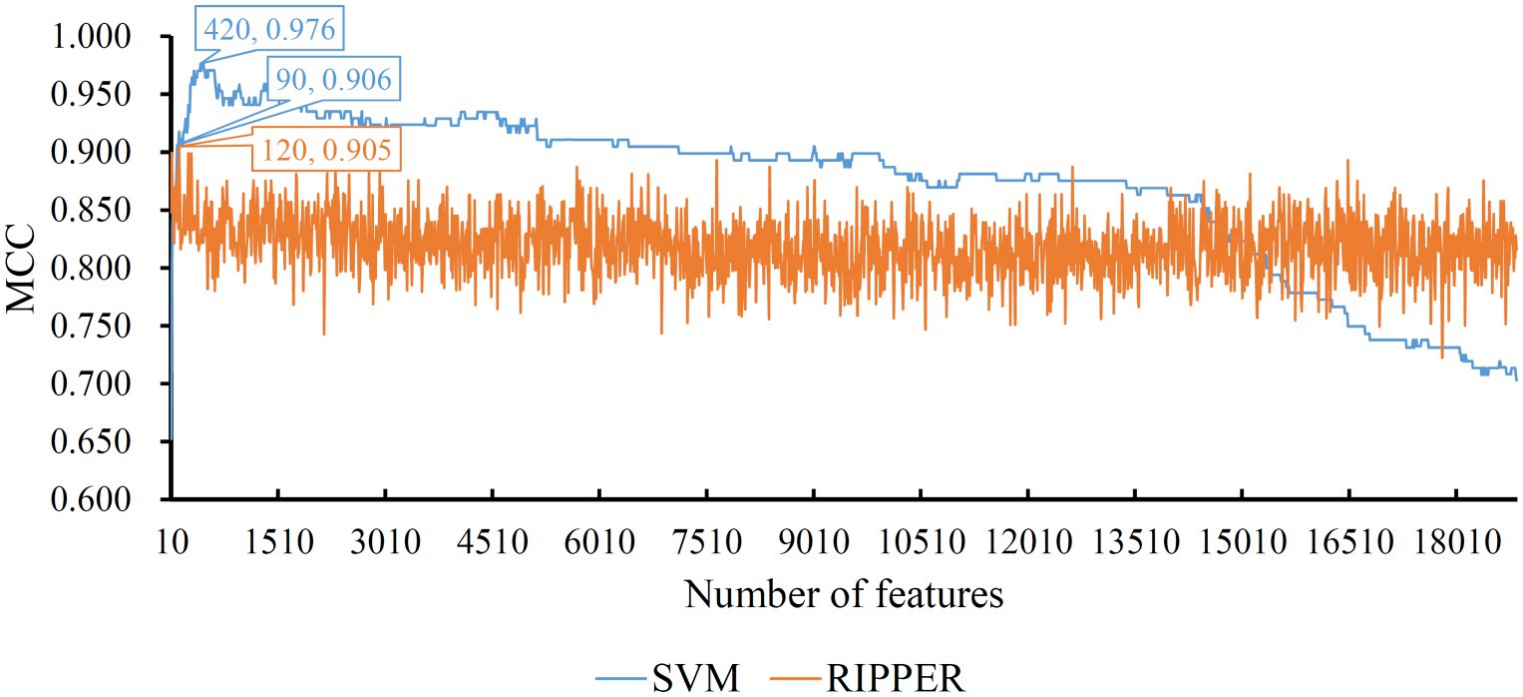
IFS curves of two classification algorithms based on feature list yielded by MCFS. The highest MCC values for SVM and RIPPER are 0.976 and 0.905, respectively, which are obtained by using top 420 and 120, respectively, features in the list. The SVM classifier using top 90 features also provides good performance.

**Fig 3:**
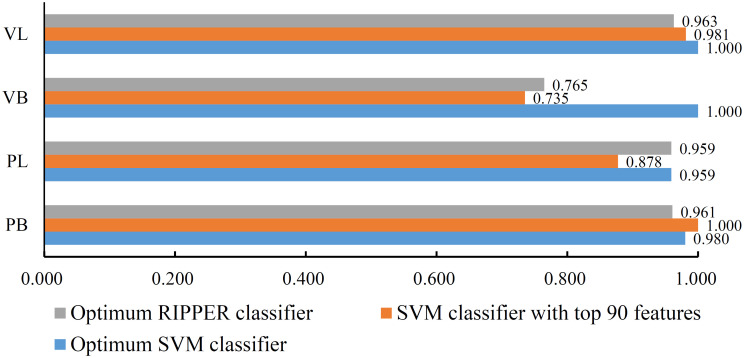
Individual accuracies of three key classifiers. The optimum SVM classifier gives quite high even perfect performance on four classes. The SVM classifier with top 90 features and the optimum SVM classifier provide almost equal performance.

The feature list yielded by the MCFS method was fed into IFS method. We first employed SVM as the classification algorithm. Several SVM classifiers were set up using different top features in the list. And these classifiers were tested by 10-fold cross-validation. Obtained measurements are listed in S2 Table. To clearly display the performance of these classifiers, an IFS curve was plotted, as shown in Fig 2, by setting MCC as Y-axis and number of features as X-axis. It can be observed that the highest MCC was 0.976. This value was obtained by a SVM classifier using top 420 features. Thus, this classifier was called optimum SVM classifier. The overall accuracy of such classifier was 0.983, as listed in Table 1. Four individual accuracies on four classes are shown in Fig 3. The classifier gave quite high performance on all classes, especially on VB and VL.

**Table 1:** Performance of some key classifiers.

Classification algorithm	Number of features	Overall accuracy	MCC
Support vector machine	420	0.983	0.976
Support vector machine	90	0.933	0.906
Repeated incremental pruning to produce error reduction	120	0.933	0.905

Although the optimum SVM classifier provided quite good performance, its efficiency was not very high due to the number of features. By carefully checking the IFS results in S2 Table, we found that when top 90 features were used, the SVM can yield the MCC of 0.906. This value was lower than the highest MCC, however, it was still acceptable. The overall accuracy of this classifier was 0.933 (Table 1). Its detailed performance on four classes is shown in Fig 3. Such classifier gave high even perfect performance on PB and VL, whereas its performance on VB was low. As this classifier used much less features, it was much more efficient than the optimum SVM classifier. It can be a fast tool to classify mammary epithelia cells.

### Results of IFS with RIPPER

As mentioned above, SVM is a black-box algorithm. It cannot provide useful clues to help us understand the mammary epithelia changes after pregnancy. In view of this, RIPPER was also employed in the IFS method. Similarly, lots of RIPPER classifiers were built and tested by 10-fold cross-validation. The measurements are available in S2 Table. An IFS curve was also plotted, as illustrated in Fig 2. The highest MCC was 0.905, which was based on top 120 features. Accordingly, the optimum RIPPER classifier was constructed with these features. The overall accuracy of this classifier was 0.933, as listed in Table 1. The detailed performance on four classes is shown in Fig 3. Evidently, the optimum RIPPER classifier was inferior to the optimum SVM classifier. However, it almost gave equal performance to the SVM classifier with top 90 features. Thus, such performance was acceptable.

### Discriminative genes and rules

Two SVM classifiers were proposed in this study. They used top 420 and 90 features, respectively. As the SVM classifier with top 90 features provided acceptable performance, top 90 features were quite essential for classifying mammary epithelia cells. Their corresponding genes were picked up as discriminative genes.

The optimum RIPPER classifier used top 120 features. With these features, we further used RIPPER to learn rules based on all cell samples represented by them. Seven rules were obtained, as listed in Table 2. Four rules were for VB and one rule was for each of other classes.

**Table 2:** Rules generated by RIPPER.

ID	Rule	Class
Rule-1	(Tbata > = 1.3033) and (Ssh2 > = 0.1463)	VB
Rule-2	(Apoe > = 1564.7779) and (Acta2 < = 6013.4274)	VB
Rule-3	(Iqub > = 0.0098)	VB
Rule-4	(Tusc5 > = 3.6569)	VB
Rule-5	(Gjb6 > = 0.0158) and (Mal2 > = 0.1745)	PL
Rule-6	(Cldn7 > = 0.8431)	VL
Rule-7	Others	PB

### Analysis of discriminative genes

Based on the 90 top ranked features, we selected seven optimal genes as examples for further discussion, which are listed in Table 3. Based on literature, these genes are widely used as biomarkers for classification.

**Table 3:** Important discriminative genes for distinguishing mammary epithelia cells.

Gene symbol	Description	RI score
Cldn7	Claudin 7	0.6574
Gjb6	Gap Junction Protein Beta 6	0.5942
Sparc	Secreted Protein Acidic And Cysteine Rich	0.5584
Cldn3	Claudin 3	0.3598
Cited1	Cbp/P300 Interacting Transactivator With Glu/Asp Rich Carboxy-Terminal Domain 1	0.3334
Krt17	Keratin 17	0.3098
Spp1	Secreted Phosphoprotein 1	0.2669

The first identified gene in our prediction list is *Cldn7*, which encodes a protein named as **Claudin 7**. Claudin 7 is a type of membrane protein that participates in the formation of tight junctions between epithelial cells. Tight junctions physically block the free diffusion of the liquids through the lacuna between cell sheets and are involved in cell and cell communications. Claudin 7 may be involved in the transportation of vesicle to the basolateral membrane, possibly stabilizing cytoplasmic vesicles or participating in cell–matrix interactions [33]. Claudin 7 plays a significant role in mammary neoplasia, and its loss or reduction in expression can result in cellular disorientation, detachment, and invasion in breast tumors [34]. In addition, the claudins, as a family of tight junction, play a specific role in mammary tumorigenesis. For example, claudin 1 and 3 are associated with mammary tumor [35]. As for its correlation with pregnancy, in 2016, researchers reported that the expression level of claudin 7 in the mammary basal cells is altered even during different stages of pregnancy and post-pregnancy period together with other pregnancy associated famous proteins, such as Connexins, E-cadherin, and β-catenin and during non-pregnancy [36]. Therefore, such predicted gene can not only distinguish basal mammary cell from luminal mammary cell but can also identify the pregnant status of the mice, corresponding with our prediction.

**Connexin 30 (*Cx30*)**, also known as gap junction beta-6 protein (*Gjb6*), is prevalent in the two distinct gap junction systems, including the epithelial cell gap junction network and the connective tissue gap network. *Cx30* has a stage-dependent expression pattern [37]. During the early stages of pregnancy, the expression cannot be detected in the mammary glands, while its mRNA and protein have strong expression in late pregnancy and lactation period. At the beginning of lactation, the expression is dramatically upregulated. Moreover, *in vitro* study on the *Cx30* expression in mouse mammary cells demonstrated its unique expression in epithelial cell, which can be affected by lactogenic hormones [37]. Therefore, *Cx30* may be essential for the development of the mammary gland. As a major ingredient of gap junctions, connexin proteins have diverse distribution. For example, *Cx43* (*Gja1*) has unique expression in myoepithelial cells, while *Cx30*, *Cx26* (*Gjb2*), and *Cx32* (*Gjb1*) are only expressed in the luminal epithelial cells [38]. The above results indicate that our selected gene *Cx30* can function as an optimal marker, because it has differential expression pattern not only in pregnancy and virgin phases of mammary cells, but also in different mammary cells, including luminal and basal epithelial cells.

**Secreted Protein Acidic And Cysteine Rich (*Sparc*)**, also known as osteonectin (*ON*), is a matricellular glycoprotein involved in the calcification process of bone collagen, wound healing, and extracellular matrix synthesis. In mammary carcinoma cells, *SPARC*, which is produced by host leukocytes instead of the tumor, shapes the stroma of the tumor [39]. *SPARC* functions as an extracellular modulator of calcium and contributes to the damage repair and proliferation in endothelial cell [40]. In comparison with normal breast, the transcript level of *SPARC* is significantly high in tumor tissue, suggesting that especially in basal cells [41], *SPARC* plays an important role in tumorigenesis [42]. Although not reported in mice, our predicted gene *SPARC* is upregulated during pregnancy in humans [43] compared with virginal status. Therefore, *SPARC*, as an up-regulated gene during pregnancy, which is mostly detected in the basal cells, contributes to the classification of different cell types at different physical stages as a potential effective biomarker.

As an effective homologue of *Cldn7* mentioned above, **Claudin 3** (*Cldn3*) is a member of the Cldn family. Cldn family consists of 24 gene members and is the major component of tight junctions. Only few data have been reported on the role of Cldn family in breast lesions, and their function has not been fully determine. The loss of CLDN expression in the majority of grade 1 invasive carcinomas suggests a special role for this protein in mammary glandular cell differentiation and carcinogenesis [44]. Moreover, claudin 1 and 3 are commonly detected in mammary tumors, and the wide distribution of claudin 3 suggests its important role in mammary physical differentiation and tumorigenesis [35]. These findings indicate that the predicted gene claudin 3 might be differentially expressed in the pregnancy and virgin phases of mammary epithelial cells. In luminal or basal cells, claudin 3 is remarkably upregulated in basal cells during pregnancy, indicating that such protein may not only distinguish different cell subtypes but also different pregnancy status [45].

Cbp/p300-interacting trans-activator 1 (***Cited1***), also known as melanocyte-specific gene 1, functions as a transcriptional coactivator. A human breast cancer dataset revealed that *CITED1* has a similar expression pattern with *STC2*, *AREG*, and *Erα*, and *CITED1* expression is always associated with good prognosis in breast cancer [46]. *Cited1* knockout mouse was constructed, and the homozygous mice showed abnormal mammary ductal morphogenesis at puberty [47]. This phenomenon occurred, because *Cited1* can affect the expression of both individual estrogen and TGFβ downstream transcriptional target genes in the pubertal gland. Estrogen is differentially detected and expressed in luminal and basal cells [48, 49]. Moreover, during different stages of pregnancy and comparing pregnant or virginal status, the expression level of estrogen also differs [50]. Therefore, considering its tight correlations with estrogen, such protein *Cited1* has different expression level between basal and luminal cells at different stages of pregnancy/virgin.

The next predicted gene ***Krt17***, also known as type I cytoskeletal 17, is a type of basal cytokeratin. A keratin protein regulates the epithelial cell growth. The Akt/mTOR signaling pathway plays an important role in the synthesis of proteins and thus regulates the cell proliferation, tissue development, and organogenesis. The simultaneous stimulation of mTOR activity and cell growth requires two amino acid residues in the 17 amino-terminal head structural domain of keratin [51]. *Krt17* is identified by supervised analysis, differentially expressed in luminal and basal cell lines, and is a type of overexpressed gene and reported basal maker [52]. Therefore, *Krt17* may be differentially expressed in basal and luminal cells. As for its differential expression level under pregnant and virginal status, the biological procedure pregnancy itself can control the epithelial composition and hormone regulation via regulating functional proliferation-associated genes, such as *KRT17*, implying that *KRT17* may also have different expression levels during pregnancy [53].

The predicted gene ***Spp1***, also known as osteopontin (*Opn*), has been observed by various human cancers, including breast cancer. It is a secreted glycophosphoric acid protein involved in mammary gland development. The expression of *Spp1* differs at different stages during mammogenesis. *Spp1* has low-to-moderate expression levels in the nulliparous gland but remarkably high expression in the lactating gland [54], suggesting that it can be used as a maker to identify differentiated epithelial cells. *Spp1* expression is crucial for mammary gland development [55]. Therefore, the *Spp1* is differentially expressed in different phases of basal and luminal cells. *Spp1* is upregulated during pregnancy, thus inhibiting cell apoptosis and promoting cell proliferation in multiple female regeneration-associated tissues [56].

The top optimal genes are differentially expressed in basal and luminal cell subtypes under pregnant and virginal status, corresponding with our prediction and validating the efficacy and accuracy of our newly presented methods.

### Functional enrichment analysis on discriminative genes

The functional enrichment analysis was also performed on top 90 feature genes to uncover biological meanings behind these genes. The results are provided in S3 Table. It can be observed that several top terms are related to cell junction organization, including GO:0120193 (tight junction organization), GO:0045216 (cell-cell junction organization), GO:0034329 (cell junction assembly), GO:0120192 (tight junction assembly), GO:0007043 (cell-cell junction assembly) and mmu04530 (Tight junction), which is consistent with the inclusion of *Cldn7*, *Cldn3*, *Cldn4*, *Cx26* (*Gjb2*) and *Cx30* (*Gjb6*) in our top ranked features and rules.

*Cldn7*, *Cldn3*, *Cldn4* and *Cldn19* all belong to the Claudin family consisting of 24 gene members. Claudin is the major component of tight junction complex, which provides a form of adhesion for epithelial or endothelial cells, and regulates cell proliferation, differentiation and maintaining cell polarity [57]. Claudins play an important role in mammary gland development. Blanchard et al. brings strong evidence that *Cldn1*, *3*, and *4* are differentially expressed in the mammary gland at different stages of mammary gland development [35]. The expression of Claudins differs in different mammary epithelial cell subtypes. *Cldn3* is remarkably upregulated in basal cells during pregnancy and *Cldn4* has a strong expression in luminal epithelial cells, indicating that such protein may not only distinguish different cell subtypes but also different pregnancy status [41, 45]. Furthermore, the previous studies have proved the aberrant expression of Claudins are associated with the malignant transformation of mammary epithelial cells [58–60]. As a member of tight junction molecules, the expression of *Cldn19* is downregulated in breast carcinomas, indicating it plays a key role in the maintenance of normal mammary gland [61].

*Cx26* and *Cx30* belong to Connexins, which can interact with Claudin-7 and participate in the transient formation of junctional nexuses in mammary gland after pregnancy, indicating that they may have different expression in the phases of virginity and pregnancy [36]. The expression of Connexins can help to distinguish basal and luminal cells. According to Mroue et al.’s study [38], *Cx43* has unique expression in myoepithelial cells, while *Cx30*, *Cx26*, and *Cx32* are only expressed in the luminal epithelial cells.

### Quantitative analysis of discriminative rules

In addition to the qualitative analysis, we also identify seven rules for quantitative analysis on the distinction of PB, PL, VB, and VL, which are listed in Table 2.

The first rule (Rule-1) involves two significant genes: thymus, brain, and testes associated (***Tbata***) and protein phosphatase slingshot homolog 2 (***Ssh2***). According to our prediction, these two genes might be highly expressed in basal mammary cells in virgin period. *Tbata* gene is mainly expressed in highly polarized cell types, including testis germ cells, brain neurons, and thymic epithelial cells (TEC [62]. Considering the mammary epithelium cell as a type of highly polarized cell, the higher expression of *Tbata* may indicate VB case (basal mammary cells in virgin). Although no confirmed reports have validated the differential expression level of *Tbata* in pregnant and virginal status at present, another candidate rule parameter *Ssh2* may contribute to the further classification. *Ssh2* plays an important role in actin dynamic, which is important for the formation of epithelial cells by reactivating ADF/confilin proteins *in vivo* [63], suggesting that the *Ssh2* gene is important for epithelial mammary cells. Further, in 2011, *SSH2* has been selected as a potential biomarker for early pregnancy prediction in peripheral blood at the mRNA level [64]. Considering the source of peripheral blood mRNA and the expression distribution patterns of *Ssh2*, in breast tissues, *Ssh2* may also have specific expression level during pregnancy, corresponding to our rule.

The identified rule (Rule-5) also involves two genes, namely ***Gjb6*** and ***Mal2***. Based on the analysis above, the higher expression of these two genes indicates that such sample may be luminal mammary cells of pregnancy. As described above, *Gjb6* is highly expressed in luminal epithelial cells in pregnancy mammary, corresponding to our prediction rules. *Mal2* encodes a multi-span trans-membrane protein, and Mal2 protein is a component of lipid rafts. ML is an essential element of the machinery in the epithelial cells. *Mal2* is correlated to the proliferation of mammary cells and also a general phenotype during pregnancy [65]. Therefore, these two genes, which are highly expressed, are potential parameters for the distinction of luminal and basal cells.

The last two rules (Rule-6 and Rule-7) are related to the gene ***Cldn7***, whose higher expression may represent the basal mammary cells in pregnancy and the luminal mammary cells in virgin. Claudin 7 is expressed constitutively in the mammary epithelium and Claudin 7 may be involved in the transportation of vesicle to the basolateral membrane [33], suggesting its participation in both two types of epithelial mammary cells and can be the feature to differentiate the epithelial cells in virgin and pregnancy mammary cells.

## Conclusion

Collectively, using machine learning algorithms, we recognized discriminative feature genes and identified some discriminative rules to classify luminal and basal cells in the phases of virgin and pregnancy. The cross-validation results suggested that they were efficient to make classification. The biomarkers (discriminative genes) and rules we identified can not only help reveal molecular profiling patterns for different mammary cells at different phases, but also set up a quantitative standard to recognize mammary cell different subtypes and different phases for further clinical applications. Our newly presented computational approach can detect potential biomarkers and determinative rules for cell identity and provide molecule foundation for further mechanism study of the different types of epithelial mammary cells and their interactions.

## Supporting information

S1 Table List of ranked features from MCFS. (XLSX)

S2 Table IFS performance with different classification algorithms and number of top features in the list yielded by MCFS. (XLSX)

S3 Table Functional enrichment analysis on discriminative genes. (XLSX)
